# Transcriptome Analysis of the Effects of Grafting Interstocks on Apple Rootstocks and Scions

**DOI:** 10.3390/ijms24010807

**Published:** 2023-01-02

**Authors:** Qingshan Li, Yuan Gao, Kun Wang, Jianrong Feng, Simiao Sun, Xiang Lu, Zhao Liu, Deying Zhao, Lianwen Li, Dajiang Wang

**Affiliations:** 1 Key Laboratory of Horticulture Crops Germplasm Resources Utilization, Research Institute of Pomology, Chinese Academy of Agricultural Sciences (CAAS), Ministry of Agriculture and Rural Affairs of the People’s Republic of China, No. 98 Xinghai South Street, Xingcheng 125100, China; 2 Xinjiang Production and Construction Corps Key Laboratory of Special Fruits and Vegetables Cultivation Physiology and Germplasm Resources Utilization, Horticulture, Agricultural College of Shihezi University, Shihezi 832003, China

**Keywords:** apple, RNA-seq, interstocks, grafting

## Abstract

Apples are a major horticultural crop worldwide. Grafting techniques are widely utilized in apple production to keep the varieties pure. Interstocks are frequently used in Northern China to achieve intensive apple dwarfing cultivation. High-throughput sequencing was used to investigate differentially expressed genes in the phloem tissues of two different xenograft systems, M (‘Gala’/‘Mac 9’/*Malus baccata* (L.) Borkh.) and B (*‘Gala’*/*Malus baccata* (L.) Borkh.). The results showed that dwarfing interstocks could significantly reduce the height and diameters of apple trees while have few effects on the growth of annual branches. The interstocks were found to regulate the expression of genes related to hormone metabolism and tree body control (*GH3.9*, *PIN1*, *CKI1*, *ARP1*, *GA2ox1* and *GA20ox1*), these effects may attribute the dwarf characters for apple trees with interstocks. Besides, the interstocks reduce photosynthesis-related genes (*MADH-ME4* and *GAPC*), promote carbon (C) metabolism gene expression (*AATP1*, *GDH* and *PFK3*), promote the expression of nitrogen (N)-metabolism-related genes (*NRT2.7*, *NADH* and *GDH*) in rootstocks, and improve the expression of genes related to secondary metabolism in scions (*DX5*, *FPS1*, *TPS21* and *SRG1*). We also concluded that the interstocks acquired early blooming traits due to promotion of the expression of flowering genes in the scion (*MOF1*, *FTIP7*, *AGL12* and *AGL24*). This study is a valuable resource regarding the molecular mechanisms of dwarf interstocks’ influence on various biological processes and transplantation systems in both scions and rootstocks.

## 1. Introduction

Grafting is an agronomic practice with a 4000-year history in China that involves the attachment of one plant’s branch or bud (scion) to the rhizome (rootstock) of another plant [1]. This agronomic strategy is mostly used to grow horticultural crops such as vegetables [2] and fruit trees [3]. The rootstock and scion interact with each other, and the rootstock can improve the scion’s growth and yield, as well as the plants’ ability to respond to biotic or abiotic stresses [4,5,6,7]. The selection of a suitable rootstock is one of the major steps to achieve excellent tree performance in different ecological regions [8].

The apple is one of the most important temperate fruit trees in China and globally [9]. The majority of customers hold it in high regard [10]. In the cultivation of apples, grafting is a key agricultural technique that is frequently used [11]. Dwarfing and dense planting, which can encourage early blooming, increased yields, and improved fruit quality, are currently in vogue [12]. Two basic types of intensive dwarfing cultivation involve the use of dwarfing rootstocks and dwarfing interstocks [13]. Dwarfing interstocks can limit the growth of scion varieties by impeding the transport of nutrition [14], hormones [15], water [16], and mineral salts [17]. A variety of rootstocks can improve apple fruit quality in terms of soluble solid contents and sugar–acid ratios [18], as well as encouraging young plants to bloom early [19] and exhibit greater cold and disease resistance [20,21]. In addition to apples, interstocks are commonly used in peach, cherry, pear, persimmon, and orange trees, several studies have reported the various influences on plant vigor, hormonal, stress tolerance to scion or rootstock of interstocks [6,7,8,9,12,13,14].

The economic effectiveness of apple cultivation depends on the wise selection and application of interstocks. Many fruit trees may benefit from a suitable interstock in terms of fruit yield, variety identification, and stress resistance [22]. Apples, bananas, pears, sweet oranges, and other fruit trees have had their whole genomes sequenced [23]. All of these factors have made it possible to study the impact of transcriptional levels on the rootstock and scion of the grafting interstock, but the biological processes by which an interstock imparts dominant traits to the scion and how the interstock and scion interact biologically are still unknown in apple trees.

Researchers have looked into how the material is exchanged between the rootstock and scion, showing that the key to the rootstock–scion interaction is recognition and information flow between rootstock and scion cells [24]. Phloem tubes contain hundreds of types of RNA, including short RNAs, noncoding RNAs and mRNAs. These RNAs can affect various aspects of plant growth, such as leaf development, fruit quality, and root formation [25,26]. The molecular biology of the dwarfing mechanism and stock interaction of apple trees has witnessed a number of advances. However, due to the complexity of the fruit tree’s genetic background, more research is needed to advance our understanding of the apple dwarfing mechanism.

The molecular mechanisms of the apple interstock interface and the mRNAs transported to and from the grafted dwarf interstock are mostly unclear. We performed RNA sequencing of the phloem from various locations in two xenograft systems: the dwarf interstock M (‘Gala’/‘Mac 9’/*Malus baccata* (L.) Borkh.) and the dwarf rootstock B (‘Gala’/*Malus baccata* (L.) Borkh.). Based on the transcriptome analysis, the potential role of grafting reaction mRNA in various biological and metabolic processes is discussed in this paper, and an excellent resource is provided for elucidating the molecular mechanism of the rootstock effects of dwarf interstock in various biological processes and transplantation systems.

## 2. Results

### 2.1. The Effect of Dwarfing Interstock on the Tree Body

**Figure 1:**
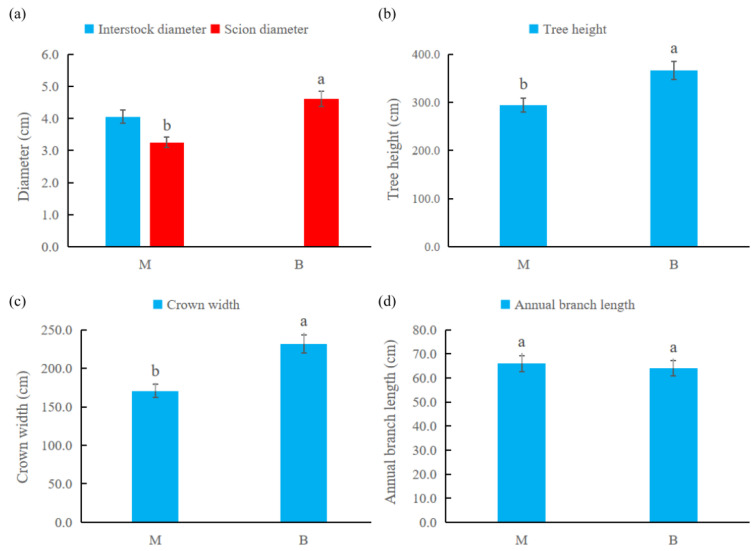
Effect of dwarf interstock on tree body growth indices. (**a**) Effect of dwarf interstock on diameter of apple tree. (**b**) Effect of dwarf interstock on height of apple tree, (**c**) Effect of dwarf interstock on grown width of apple tree. (**d**) Effect of dwarf interstock on annual branch length of apple tree. (Significant differences between values in the same row are indicated by different lowercase letter superscripts (*p* < 0.05)).

The effect of dwarfing interstock on tree development and growth has been found to be directly reflected in the growth strength of trees [22]. Comparing the growth of the B and M combinations (Figure 1), the tree height and crown width of M were significantly less than those of B, as was the scion diameter. However, the annual branch length did not differ significantly between B and M. The annual branch growth was hindered after interstock grafting. This had a negative impact on the development of the tree, resulting in tree dwarfing.

### 2.2. Quality Control of Transcriptome Data

Five phloem samples from B and M combination were used to create the library. After filtering the raw data, a total of 116.49 GB clean data was obtained, with each sample comprising 6.56 GB. The average of Q30 was 90.94%, with an average GC content of 47.57%. Clean reads from each sample were sequenced against the reference genome, with alignment efficiency ranging from 80.40% to 87.17% (Table S1).

**Figure 2:**
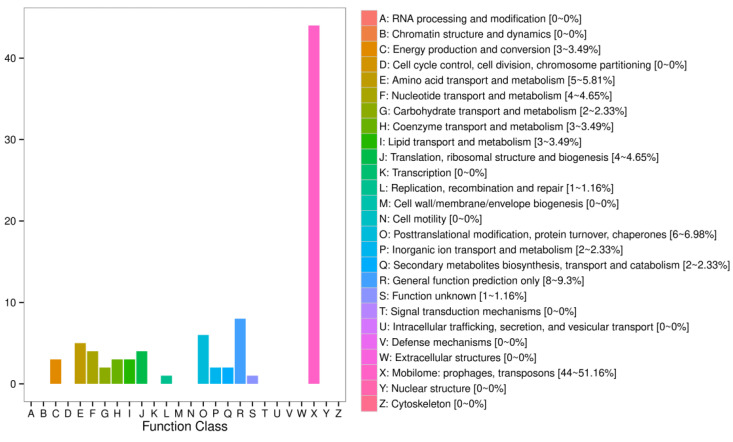
COG analysis of the new genes. (X-axis: the COG functional classifications. Y-axis: the new genes number in each COG functional classifications. On the right are listed the functional categories corresponding to each number, the number of new genes in this category and the proportion of the total number of new genes).

A total of 5286 new genes were identified, with 2851 of them annotated in various databases (Table S2), including COG (87), GO (1088), KEGG (855), KOG (347), Swiss-Prot (339) and nr (2808). The top three COG function classes were mobilome: prophages and transposons (44, 51.16%); general function prediction only (8, 9.30%); and posttranslational modification, protein turnover, and chaperones (6, 6.98%) (Figure 2).

### 2.3. Analysis Expression Profiling of Differentially Expressed Genes (DEGs)

**Figure 3:**
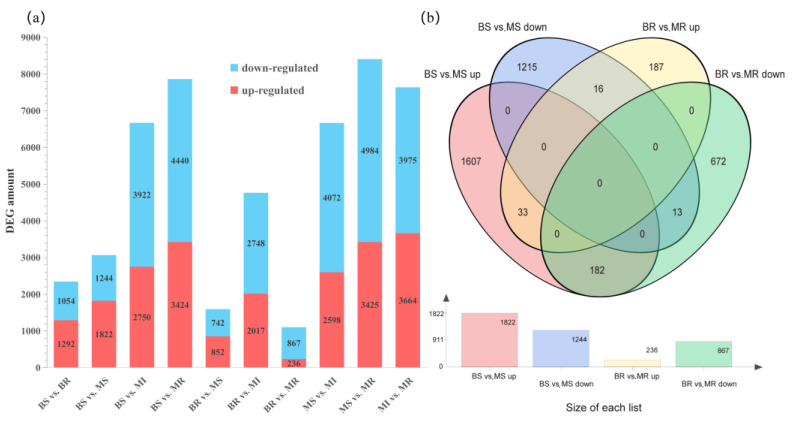
Analysis of DEGs. (**a**) Number of DEGs in each group. (**b**) Venn diagram analysis of DEGs (different colors represent different comparison combinations).

During the detection of DEGs, fold change ≥ 2 and FDR < 0.01 were used as the screening thresholds, and the significance *p*-values obtained from the original hypothesis testing were corrected. We detected DEGs by comparing five pairs of samples (Figure 3a). A total of 15,555 DEGs were identified (Table S3). The comparison of BS vs. MS (B scion vs. M scion) revealed 1822 upregulated genes and 1244 downregulated genes. BR vs. MR (B rootstock vs. M rootstock), in contrast, had 1103 DEGs, of which 236 genes were upregulated and 867 genes were downregulated. The DEGs were subjected to cluster analysis, and a cluster heat map was created (Figure S1a,b). The expression levels of the genes in the group’s three replicates tended to be roughly consistent, indicating that biological reproducibility was reliable. We analyzed the DEGs of the BS vs. MS and BR vs. MR combinations using Venn diagrams (Figure 3b). The interstock had more effects on the scion than on the rootstock. A total of 182 genes were upregulated in the scions but downregulated in the rootstocks. A total of 16 genes were downregulated in the scions but upregulated in the rootstocks. Additionally, after grafting the interstock, there were 33 DEGs that were commonly upregulated in the BS vs. MS and BR vs. MR groups and 13 DEGs that were commonly downregulated in these groups (Figure 3b) (Table S4). We focused on DEGs that were commonly upregulated or downregulated in the scions and rootstocks under grafted interstocks. These genes were found primarily to relate to the transport (ABC transporter G family member 14 (*ABCG14*)), plant stress resistance (cytochrome P450 78A5 (*CYP78A5*) and disease resistance protein RPP13)), and hormone metabolism areas (auxin-responsive protein SAUR36).

### 2.4. GO Function Analysis of DEGs

GO enrichment analyses were used for the BS vs. MS and BR vs. MR DEG analyses, and it was revealed that these DEGs were primarily focused on three process groups: biological processes (BPs), cellular components (CCs), and molecular functions (MFs) (Figure S2). The major GO terms for BS vs. MS were metabolic processes, cellular processes, and single-organism processes for BPs; the major GO terms in the membrane and membrane part were CC; and the major GO terms in MFs were binding and catalytic activity. In BR vs. MR, the major GO terms were metabolic processes and single-organism processes for BP; the major GO terms in the cell, cell part, membrane, and membrane part were CCs; and major GO terms in the MFs were binding and catalytic activity.

**Figure 4:**
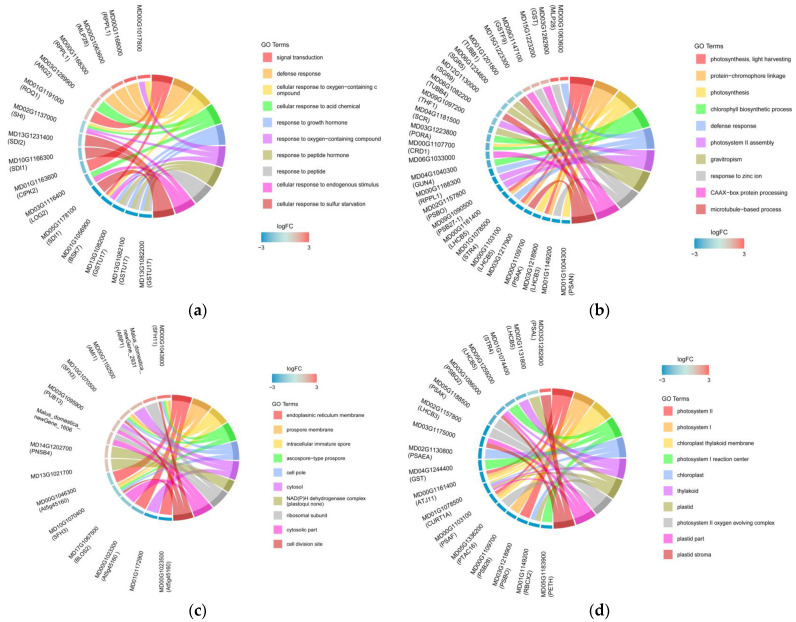

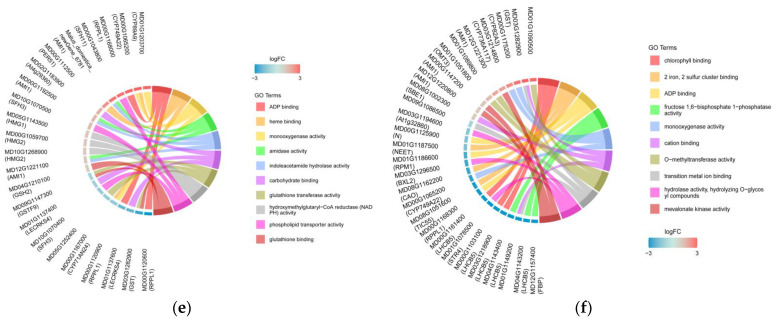
Enrichment string diagram of three DEGs from the ten most significantly enriched GO terms. (**a**,**b**) Biological process enrichment string diagram of BS vs. MS and BR vs. MR, respectively. (**c**,**d**) Cellular component enrichment string diagram of BS vs. MS and BR vs. MR, respectively. (**e**,**f**) Molecular function enrichment string diagram of BS vs. MS and BR vs. MR, respectively. (DEG IDs (name) on the left, each gene’s corresponding color is log_2_FC; the GO terms functional annotation on the right, different colors represent various GO terms).

Simultaneously, we used the *q*-value to assess the significance of the GO terms. The top 20 GO terms with high significance in the BS vs. MS group are shown in Figure S3a–c, and those in the BR vs. MR group are shown in Figure S3d–f. In BS vs. MS, the top three BP terms were signal transduction, defense response, and cellular response to oxygen-containing compounds. The top three CC terms were endoplasmic reticulum membrane, prospore membrane, and intracellular immature spore. The top three MF terms were ADP binding, heme binding, and monooxygenase activity. Photosynthesis, protein–chromophore linkage, and photosynthesis were the top three BP terms in MR vs. BR. Photosystem II, photosystem I, and chloroplast thylakoid membrane were the top three CC terms. The top three MF terms were chlorophyll binding, two iron–two sulfur cluster binding, and ADP binding. These GO term genes were all downregulated in the grafted interstock combinations, implying that grafting interstock may have an effect on the expression of photosynthesis and carbon metabolism genes in the tree body. Three genes from the ten most significantly enriched pathways were chosen for mapping in order to better mine the enriched genes and the corresponding GO terms (Figure 4a–f, Table S5). In MS, most of the upregulated expressed genes were related to plant resistance, growth, and development, such as glutathione synthetase (*GSH*), putative disease resistance RPP13-like protein 1 (*RPPL1*), auxin-repressed protein 1 (*ARP1*), and glutathione S-transferase (*GST*). In MR, genes associated with photosynthesis were mostly downregulated, such as oxygen-evolving enhancer protein 1 (*PSBO*) and chlorophyll a-b binding protein 3 (*LHCB3*).

### 2.5. KEGG Function Analyses of DEGs

**Figure 5:**
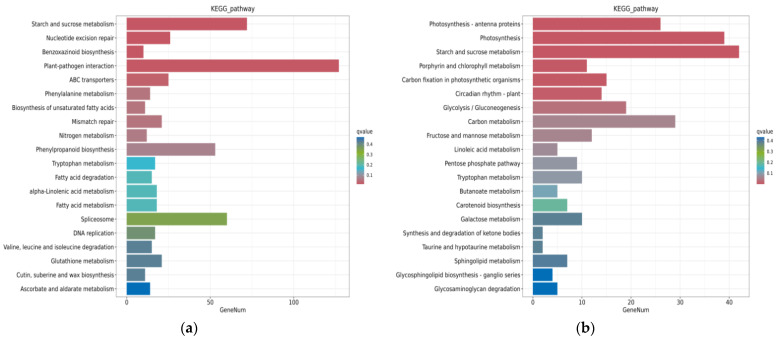
Enrichment analysis of KEGG. (**a**) BS vs. MS KEGG enrichment histogram. (**b**) BR vs. MR KEGG enrichment histogram. The ordinate is for each pathway entry, and the abscissa is the number of genes, i.e., the number of differential genes annotated in this entry. The colors in the columns represent the hypergeometric test *q*-values.

KEGG enrichment analysis was performed on the two rootstock and scion combinations (Figure S4a,b). In BS vs. MS, the KEGG pathway enrichment analysis revealed enrichment in 125 pathways. The top 20 pathways with the most reliable enrichment significance were chosen and displayed in a histogram (Figure 5a). The most significantly enriched pathways included starch and sucrose metabolism (72 DEGs), nucleotide excision repair (26 DEGs), benzoxazinoid biosynthesis (10 DEGs), plant–pathogen interaction (127 DEGs), and ABC transporters (25 DEGs). These mainly related to carbohydrate metabolism and secondary metabolites, for example, beta-glucosidase 45 (*BGLU45*), sucrose synthase 2 (*SUS2*), alpha-1,4 glucan phosphorylase L isozyme (*PHO1*), and *SRG1*. N metabolism and phenylpropanoid-biosynthesis-related genes were also enriched in the scions.

Furthermore, a total of 110 pathways were enriched in BR vs. MR. The top 20 pathways were chosen and displayed as a histogram (Figure 5b, Table S6). Photosynthesis–antenna proteins, photosynthesis, starch and sucrose metabolism, porphyrin and chlorophyll metabolism, and carbon fixation in photosynthetic organisms were the top five significant enriched KEGG pathways, with 26, 39, 42, 11, and 15 DEGs, respectively. These pathways are associated with growth, development, and secondary metabolism. In total, 133 genes were involved, including ferredoxin-1, chloroplastic (*SEND33*), chlorophyll a-b binding protein 3 (*CAB3*), malate dehydrogenase (NADP) (*MDH1*), and glyceraldehyde-3-phosphate dehydrogenase B (*GAPB*).

### 2.6. Functional Enrichment Analyses of Tissue-Specific Modules Using WGCNA

**Figure 6:**
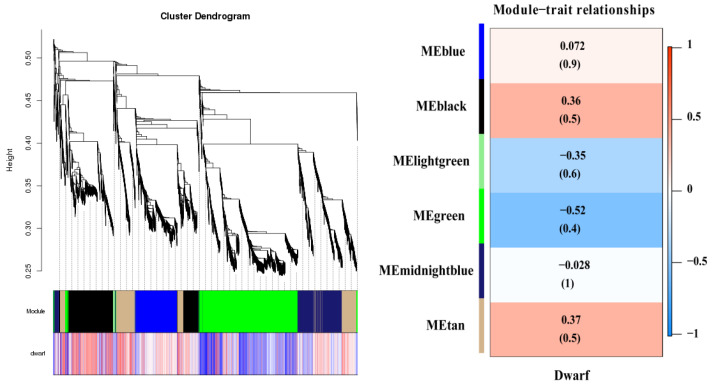
WGCNA analysis categorizes differentially expressed genes into six distinct gene modules and produces clustering results. (Analysis of the relationship between modules and dwarf phenotypes, numbers indicate correlation coefficients (kME) and *p*-values.).

The WGCNA (weighted gene correlation network analysis) approach searches for genes with significant correlations in tissues [27]. The WGCNA approach was used to identify genes highly correlated with dwarf apple trees, which were then investigated for associations with each experimental variable (scion, interstock, and rootstock genotype). A total of six modules (black, blue, green, light green, midnight blue, and tan, respectively) were screened out (Figure 6) (Table S7). kME values > 0.7 were used to identify genes with significantly correlated expression patterns, and 4627 DEGs were censored and selected (Table S8). We concentrated on MEblack, MEtan, and MEgreen in the apple dwarf trait modules. Among these, the MEblack and MEtan modules had positive correlations, whereas MEgreen had a negative correlation. The relevant genes of the three modules were analyzed using KEGG (Figure S5). MEblack contained 1232 genes; 929 genes had kME values greater than 0.7 and were concentrated in the KEGG terms for starch and sucrose metabolism, plant hormone signal transduction, phenylpropanoid biosynthesis, the MAPK signaling pathway, and carbon (C) metabolism. MEtan consisted of 914 genes, 722 of which were kME > 0.7 and were concentrated in the KEGG terms for starch and sucrose metabolism, plant hormone signal transduction, the MAPK signaling pathway, glutathione metabolism, phenylpropanoid biosynthesis, and C metabolism. MEgreen was the co-expression module with the highest negative correlation with dwarfing. It had 2040 genes, 1552 of which were kME > 0.7 and were concentrated in the KEGG terms for carbon metabolism, photosynthesis, starch and sucrose metabolism, carbon fixation in photosynthetic organisms, plant hormone signal transduction, the MAPK signaling pathway, flavonoid biosynthesis, and nitrogen (N) metabolism.

According to the WGCNA, the gene expression of the interstock had an effect on the gene expression of the scion or rootstock genome. We found that the molecular mechanism of tree body dwarfing was mainly reflected in the plant hormone metabolism, N metabolism, C metabolism, and photosynthesis genes of the scions and rootstocks. The interstock also affected the expression difference of genes in secondary metabolism, such as some genes related to phenylpropanoid biosynthesis and flavonoid biosynthesis. We examine the precise effects of interstocks on scions and rootstocks in the sections below.

#### 2.6.1. Analysis of Plant Hormone Metabolism Affected by Grafting Interstock

In this study, 108 plant-hormone-related DEGs were identified (Table S9a,b). The DEGs involved abscisic acid (ABA), auxin (IAA), brassinosteroids (BRs), cytokinins (CTKs), ethylene (ETH), gibberellin (GA), and jasmonate (JA). In MS, the following genes were upregulated: ABA-related genes: abscisic acid receptor *PYL4*; IAA-related genes: auxin-responsive protein *SAUR32*, *SAUR36*, auxin-responsive protein *IAA3*, *IAA26*, and putative indole-3-acetic acid-amido synthetase *GH3.9*; CTK-related genes: cytokinin-independent 1 (*CKI1*), Arabidopsis histidine kinase 3 (*AHK3*), and two-component response regulator *ARR18*; ETH-related genes: ethylene-responsive transcription factor 1B (*ERF1B*), *ERF095*, ethylene receptor 2 (*ETR2*), and ethylene response sensor 1 (*ERS1*)*;* GA-related genes: chitin-inducible gibberellin-responsive protein 1 (*CIGR1*), gibberellin 2-beta-dioxygenase 1 (*GA2ox1*), and gibberellin 20 oxidase 1 (*GA20ox1*); JA-related genes: *TIFY10A*, *MYC3*, and *MYC4;* and BR-related genes: serine/threonine-protein kinase *BSK5*. The main function of the upregulated genes, such as *MYB4* and more flower 1 (*MOF1*), is to encourage more flowering in plant bodies. The CTK-related histidine-containing phosphotransfer protein 1 (*AHP1*) gene and the GA-related DELLA protein *RGA* and gibberellin receptor *GID1C* genes were downregulated in MS. The primary function of the downregulated hormone metabolism genes, for example, carboxylesterase 15 (*CXE15*) and KAN1, is to regulate plant growth and development. Simultaneously, we found 22 DEGs involved in plant hormone metabolism in the BR vs. MR group, but only three DEGs were upregulated in MR. The IAA-related auxin transporter-like protein 2 (*LAX2*), auxin efflux carrier component 1 (*PIN1*), *LAX3*, and *SAUR36* genes and the GA-related probable carboxylesterase 15 (*CXE15*) gene were downregulated in MR.

#### 2.6.2. Analysis of DEGs Related to Photosynthesis

The BS vs. MS analysis showed that grafting altered the expression of 16 genes involved in the reactions of photosynthesis. A total of 10 DEGs were upregulated, and 6 DEGs were downregulated. Certain carbohydrate-transport- and -metabolism-related genes were upregulated, such as fructose-bisphosphate aldolase 1 (*FBA1*) and ribose-5-phosphate isomerase 2 (*RPI2*). NADP-dependent malic enzyme 4 (*NADP-ME*4) and glyceraldehyde-3-phosphate dehydrogenase (*GAPC*) were both found to be downregulated in MS; they are primarily involved in energy production and conversion. The BR vs. MR analysis showed that grafting altered the expression of 54 genes involved in photosynthetic reactions. All these DEGs were downregulated, including ATP synthase gamma chain (*ATPC*), photosystem I reaction center subunit VI-2 (*PSAH2*), and fructose-1,6-bisphosphatase (*FBP*). These genes may influence photosynthesis and carbon fixation in plant bodies, which in turn influences tree growth.

#### 2.6.3. Analysis of DEGs Related to C Metabolism

Interstock grafting also influenced genes involved in C metabolism in the scion and rootstock. A comparison of BS vs. MS showed 32 DEGs related to C metabolism; 18 DEGs were upregulated, and 14 DEGs were downregulated. In BR vs. MR, 11 DEGs with C metabolism in scions were downregulated upon grafting of the interstock, but only 3 DEGs were upregulated. The functions of the upregulated genes, such as glycine dehydrogenase (decarboxylating) (*GDCSP*), ATP-dependent 6-phosphofructokinase 3 (*PFK3*), glutamate dehydrogenase (*GDH*), AAA-ATPase ASD (*AATP1*), and cytosolic enolase 3 (*ENO3*), were mainly related to carbohydrate transport and metabolism. Downregulated genes, such as acyl-coenzyme A oxidase 4 (*ACX4*) and glycine, serine, and threonine metabolism (*GDCSH*), mainly related to amino acid and lipid transport and metabolism.

#### 2.6.4. Analysis of DEGs Related to N Metabolism

The interstock also influenced genes involved in N metabolism in the scion and rootstock. Genes related to N metabolism in the scions were upregulated upon grafting of the interstock. Nitrate reductase (*NADH*) and high-affinity nitrate transporter 2.7 (*NRT2.7*) were upregulated in MS but downregulated in BS. Interestingly, *GDH* mentioned above also participated in N metabolism, it was upregulated in MS. In the rootstock, genes involved in N metabolism, such as glutamine synthetase nodule isozyme (*GS1*), were downregulated

#### 2.6.5. Analysis of DEGs Related to Secondary Metabolism

We focused on analysis of the genes associated with flavonoid biosynthesis. BS vs. MS and BR vs. MR revealed 27 and 8 DEGs related to secondary metabolism, respectively. In MS, 1-deoxy-D-xylulose-5-phosphate synthase (*DX5*), farnesy l diphosphate synthase1 (*FPS1*), terpene synthase 21 (*TPS21*), affeoyl-CoA O-methyltransferase (*AMT*), cinnamyl alcohol dehydrogenase 9, strictosidine synthase, and senescence-related gene 1 (*SRG1*) were upregulated. Phenylalanine ammonia-lyase (*PAL2*) and aldehyde dehydrogenase 22a1 (*ALDH22a1*) were downregulated. In MR, the gene encoding terpene synthase 21 (*TPS21*) was upregulated, while genes encoding phytoene synthase (*PSY*) and naringenin-chalcone synthase were downregulated. In general, the interstocks can enhance the expression of flavonoid-related genes in apple trees.

### 2.7. Visualization of Transcription Factors with Grafted Interstocks

**Figure 7:**
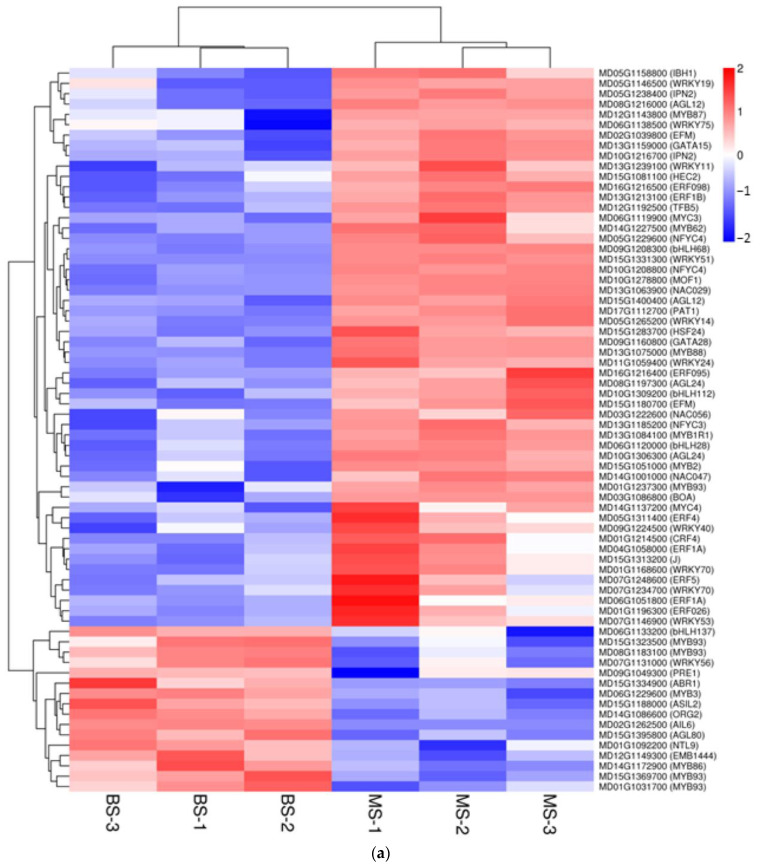

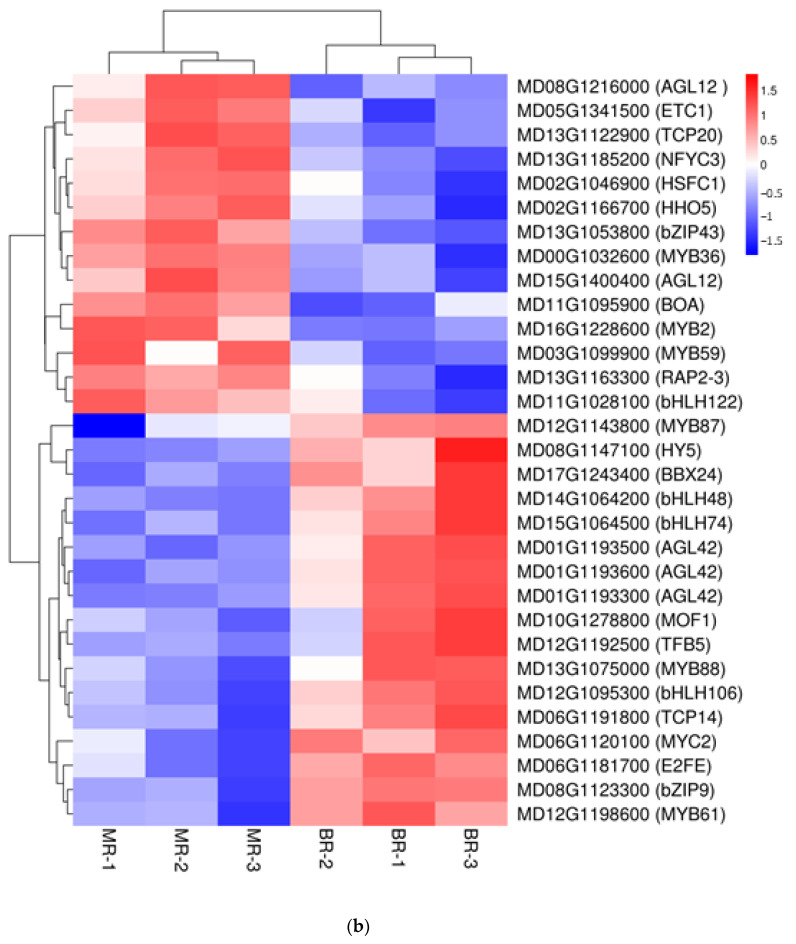
Heat map of differentially expressed transcription factors with grafted interstocks. (**a**) Differentially expressed transcription factors of BS vs. MS. (**b**) Differentially expressed transcription factors of BR vs. MR (heat map color indicates normalization of FPKM values).

In the comparison between BS and MS, we identified 70 DEGs related to transcription factors, with *MYB*s, *WRKY*s, and *ERF*s ranking as the top three types with 19, 11, and 9 DEGs, respectively. In the scion, 54 DEGs were upregulated in MS, while 16 DEGs were downregulated (Figure 7a and Figure S6). Upregulated transcription factors were mainly related to flowering regulation (agamous-like 12 (*AGL12*, *AGL24*, and *TCP2*)), plant hormone signal transduction (*EFM*, *HEC2*, *MYC3*, *MYC4*, *ERF095*, *ERF1B*, *BOA*, *MOF1*, *IPN2*, and bHLH28), and abiotic stress (*HSF24*, *MYB1R1*, *WRKY70*, *NAC025*, and *PAT1*). Downregulated transcription factors were related to flavonoid metabolism (*bHLH137*, *MYB86* and *MYB93*) and growth and development (*WRKY56*). BR vs. MR revealed 31 DEGs, with 17 downregulated and 14 upregulated genes in MR (Figure 7b and Figure S6). In MR, 10 *MYBs*, 5 *SRFs*, and 4 *bHLHs* were differentially expressed. Upregulated transcription factors were mainly related to flowering regulation (*TCP20* and *AGL12*), plant hormone signal transduction (*HHO5* and *BOA*), and abiotic stress (*HSFC1*). Downregulated transcription factors were related to growth and development (*bHLH106*, *TCP14* and *AGL42*).

### 2.8. Identification of the Mobile mRNAs with Grafted Interstocks

**Figure 8:**
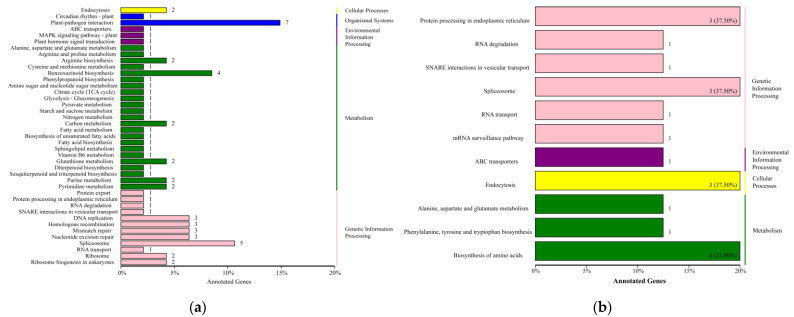
KEGG pathway enrichment analysis of the DEGs. (**a**) Classification of DEGs with KEGG pathway annotations of BS vs. MS. (**b**) Classification of DEGs with KEGG pathway annotations of BR vs. MR. (Y-axis: name of KEGG pathway annotation; X-axis: number and percentage of genes involved in this pathway over counts of all annotated genes).

In order to better understand the impacts of the interstock on the scions and rootstocks, we focused on the expression of mobile mRNAs from the interstock in the scion or the rootstock. The mRNAs were not expressed in BS but were expressed in MI and MS; these were speculated to be transmitted via the phloem to the scion. Similarly, the mRNAs specifically expressed in MR and also expressed in MI, but not expressed in BR, were considered to be delivered by the interstock to the rootstock. A total of 162 mRNAs may be transported into the scions, and 32 mRNAs may be transported toward the rootstock (Table S10). The number of these potentially mobile mRNAs delivered to the scions was greater than that delivered to the rootstocks. These potentially mobile mRNAs were subjected to KEGG enrichment analysis, and the KEGG pathway enrichment plot is shown in Figure 8. Mobile mRNAs were enriched in plant–pathogen interactions and benzoxazinoid biosynthesis in the scions. Protein processing in endoplasmic reticulum endocytosis and the spliceosome was significantly enriched in rootstocks. These genes may play a role in tree defense and sensing. Additionally, RNA transporters (eukaryotic translation initiation factor 3 subunit B (*TIF3B*) and polyadenylate-binding protein 6 (*PAB6*)) and ABC transporters (*ABCG14* and *Os02g0190300*) were among the mobile mRNAs. *TIF3B* and *PAB6* may affect RNA processing, modification, and transport. *ABCG14* and *Os02g0190300* may be related to secondary metabolite biosynthesis, transport, and catabolism.

### 2.9. qRT-PCR Validation

**Figure 9:**
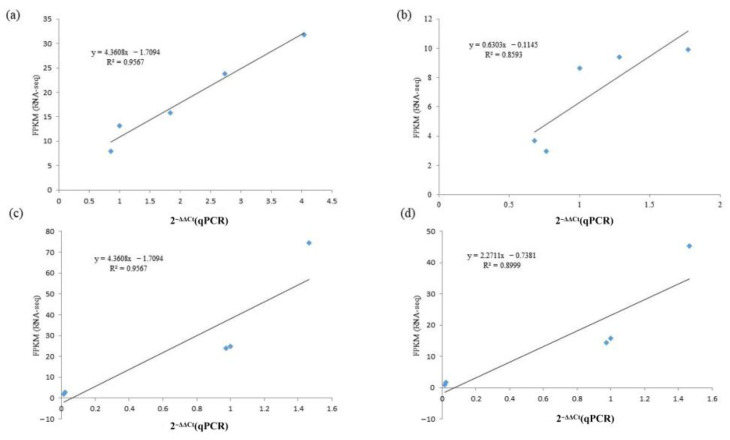
The linear relationship between RNA-seq and qRT-PCR data ((**a**–**d**) are *AGL12*, *NCED1*, *AGL24* and *NADH*, respectively).

To confirm the accuracy of our identification of the sequencing results with different rootstock–scion combinations, four random genes were selected for experimental validation using qRT-PCR. The primers were designed based on sequences from both ends of four genes. This showed that four randomly selected genes all showed expression patterns consistent with the RNA-seq results (Figure 9). The R^2^ values for the RNA-seq vs. qRT-PCR results were 0.9567, 0.9567, 0.8999 and 0.8593 for *AGL12*, *AGL24*, *NADH* and *NCED1*, respectively. These outcomes demonstrated the dependability of the genes discovered in the RNA-seq datasets.

## 3. Discussion

The rootstock, as the foundation for grafting fruit trees, influences growth trends, physiological characteristics, stress resistance, apple fruit quality, and other factors. Additionally, the scion influences the rootstock’s growth and development [4,5,6,7]. Interstocks could control scion growth, for example, restricting plant height, crown size, and tree volume [28,29]. Many previous studies have been conducted on the molecular mechanisms of the effects of dwarfing rootstocks on scions. However, the molecular mechanisms involved in dwarf interstock processes remain largely unknown. ‘Mac9’ is a popular dwarf rootstock around the world. It was bred by Michigan State University (USA) and is also known as ‘Mark’ [30]. In this research, we aimed to determine the effects of grafting interstocks on the apple rootstock and scion. The combination of the grafted ‘Mac9′ interstock resulted in more dwarfing than the vigorous stock combination, while having little effect on annual branch growth.

This might be due to the dwarfing interstock combination having two grafting interfaces, wherein each of these interfaces may result in a communication barrier for nutrients, water, and assimilates because of aberrant vascular tissue on the grafting surface. The ability of the tree body to grow is simultaneously influenced by the exchange of nutrients and hormones between the scion and the interstock, and the material metabolism between the rootstock and the interstock [31]. The interstock influences root growth and N metabolism by altering carbohydrate metabolism, auxin, and cytokinin signaling between the rootstock and scion combinations, resulting in tree dwarfing [32]. Precocious flowering in apple trees has been found to often be associated with a smaller tree size; dwarfing interstocks make the transition to flowering of apple trees easier [33].

**Figure 10:**
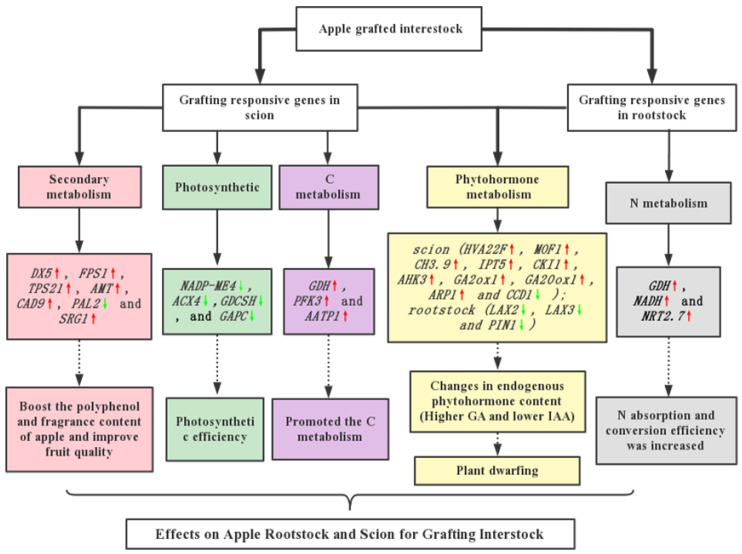
Putative working model for grafted interstock in apple trees. Red arrows: upregulated. Green arrows: downregulated.

The impact of the interstocks on the scions and rootstocks in this research was concentrated on secondary metabolism, photosynthesis, N metabolism, and phytohormone metabolism. Based on the results, we were able to propose a putative working model for the grafted interstock in apple trees by exploring the mechanisms of interaction between the rootstock, interstock, and scion (Figure 10).

Phytohormones are important for vegetative and reproductive growth [34,35]. They are signal molecules that induce tissue differentiation above and below the graft junction [36]. Phytohormones can also influence rootstock-mediated vigor by altering gene expression in the scion [37]. Previous research suggests that a change in endogenous hormone content is the primary factor determining tree size. IAA, ZR and GA3, which promote growth of plant, were inhibited in apple trees under grafting dwarfing interstocks. While ABA was promoted by interstocks in apple trees [15,31]. Model plant studies have shown that GA is primarily responsible for plant height regulation, and mutants lacking GA or insensitive to GA are short [38]. Disruptions in GA metabolism were found to play a critical role in scion dwarfing, with lower levels of GA more likely to result in dwarfing [39]. GA20ox are responsible for the degradation of GA. GA2ox play a role in inactivating gibberellins [40]. Upregulated expression of GA2ox can exhibit shorter internodes, reduce stem elongation, and decrease the amount of GA in scions [41]. In this study, the GA20ox2 and GA2ox1 genes were found to influence grafted tree growth and development by regulating gibberellin synthesis and metabolism. Their high expression may be one of the reasons for the tree dwarfing caused by interstocks; this is consistent with previous reports [42].

The ability of dwarf rootstocks to cause dwarfing may be related to IAA [43,44]. Dwarfing interstocks can cause trees to dwarf, which is related to the oxidation or degradation of auxin in the phloem [15]. In this study, the expression of some auxin transport-related genes (*LAX2* and *LAX3*), and IAA transporter gene *PIN1* was downregulated. The downregulated expression of *LAX2*, *LAX3* and *PIN1* may reduce the IAA content in scion, and cause tree dwarfing [44,45]. FT-interacting protein 7 (*FTIP7*) and *AGL12* were upregulated in the scion under the influence of the interstock. *AGL12* has been identified as a possible transcriptional activator that controls cell proliferation in the root meristem through regulation of root development. It may mediate the IAA response in roots. It can be used as a flowering transition promoter by upregulating the suppressor of the overexpression of CO1 (*SOC*), flowering locus T (*FT*), and LEAFY (*LFY*). Trees with higher levels of ABA are more likely to be dwarfed. Additionally, phytohormone synthesis and signaling are central to the molecular biology of tree body dwarfing. The expression of the IAA transporter gene *PIN1* may be related to tree body dwarfing [44,45]. The CTK-metabolism-related gene adenylate isopentenyltransferase 5b (*IPT5b*) was found to be expressed differently in different dwarf types [46]. Its overexpression can directly inhibit canassin steroid synthetase expression, thereby mediating apple dwarf stock M26 dwarfing [47]. At the scion, a limited supply of cytokinin may modify the architecture by decreasing branching, whereas a limited supply of gibberellins may primarily reduce the duration of shoot extension growth [43]. In conclusion, after interstock grafting, CTK-related genes, ABA-related genes, and GA-related genes were upregulated; IAA-related genes were downregulated; and the synergistic effect of these phytohormones caused the plant to the dwarf.

Plant photosynthesis is the foundation of all other physiological activities, ensuring organic matter accumulation and normal plant growth and development [48,49]. Carbon fixation in photosynthesis, as well as related genetic changes in N metabolism, can impact photosynthetic efficacy, subsequently influencing plant growth and development [50,51,52]. Previous studies have found that the combination of different rootstocks and scions has a significant impact on the photosynthetic rate of leaves. The remarkable role of photosynthesis in regulating plant growth is through its influence on scion growth. The ability of interstocks to impact photosynthesis was found to yield the opposite results. The use of interstocks reduced photosynthesis and the root–shoot ratio, but increased carbohydrate availability in scions in ‘Fuji’ trees [53]. However, when compared to the rootstock, the interstock significantly increased the net photosynthetic rate (Pn) in ‘Yanfu’ [29]. In this study, we discovered that the interstock had multifaceted effects on the expression of scion photosynthesis-related genes, inhibiting the expression of photorespiration-related genes while promoting the expression of genes encoding photosynthetic light reactions and the Calvin cycle. The interstock increased *AATP1* and *GDH* in the scion but inhibited the expression of the photosynthesis-related genes in the rootstock, such as *NADP-ME*4 and *GAPC*. GDH was found to be able to improve plants’ carbon fixation capacity and shorten plants’ vegetative growth cycles, which promotes the Calvin cycle and increases plant carbohydrate accumulation, resulting in higher fruit quality [54,55]. *AATP* enhances carbon metabolism and starch formation in plants by acting as an essential protein for transporting ATP/ADP on plastids [44]. *NADP-ME*4 can speed up the supply of CO_2_ to the photosynthesis-related rubisco enzyme [45]. In general, it has been shown that interstocks have a negative influence on photosynthesis in scion, the interstock may be utilized to inhibit apple photosynthesis to reduce the tree vigor. N is a necessary nutrient for plant growth and development, as well as an important component of nucleic acids, proteins, and chlorophyll [56]. Trees grafted onto dwarfing rootstocks were found to have higher N concentrations than vigorous rootstocks [57]. Using transcriptome analysis, we discovered that the interstock promoted the expression of genes involved in N metabolism. It might be influence crop growth and development, as well as fruit quality, while also increasing plant resistance to adversity. The enzymes *NR* and *GDH* were found to be important in the N metabolism process in plants. *NR* can convert NO^3−^ absorbed from the soil in the cytosol to NO^2−^, which can then be processed and utilized downstream [58]. *GDH* plays an important role in carbon and N metabolism [59]. Additionally, the transcription of root N-metabolism-related genes and the concentration of N metabolites were found to be the most important regulators of plant growth [60]. In this study, interstock might be increased N absorption and conversion efficiency. The upregulation of *GDH* and *NRT2.7* expression in the rootstock may be an important factor when grafting interstocks to improve tree stress resistance and fruit quality.

Fruit quality was found to often be higher when grafting interstocks in apple trees [61]. Interstocks can not only increase fruit yield [15,62], the content of soluble solids, and the glycan acid ratio, but can also increase the contents of fruit secondary metabolites such as polyphenols and aromatic substances [30,63,64]. After grafting the interstock, most phenylpropanoid-, flavonoid-, and isoprenoid-related genes were found to be upregulated in the scion, such as FPS1, TPS21 and AMT. DX5 was found to be a rate-limiting enzyme in the Embden–Meyerhof–Parnas (EMP) pathway, which regulates the biosynthesis of various types of terpenoids [65], and FPS1 is a key enzyme in the terpenoid synthesis pathway [66]. The upregulation of DXS and FPS1 expression in the scions may be one of the means by which the interstock is able to improve fruit aroma substances. Dihydroflavonol 4-reductase (DFR) was found to be a key enzyme that catalyzes the conversion of dihydroflavonol to anthocyanin precursors. It determines the types and content of anthocyanins to some extent [67]. Upregulation of two DFR genes in MS may increase the polyphenol content of the fruit, promoting fruit coloration. We discovered that interstock might increase polyphenol and aroma related gene expression and improve fruit quality. Dwarfing interstocks have been shown in numerous studies to shorten the juvenile phase, increase early yield, and promote early flowering in fruit trees [18,33]. Interstocks can weaken the tree’s potential and dwarf the tree’s body by reducing the number of long branches and increasing the number of flowers [68]. We discovered that the flowering genes AGL12, AGL24, RAX3 and REM20 were significantly upregulated in the scions, which may result in early tree flowering.

## 4. Materials and Methods

### 4.1. Experiments with Planting Materials and Grafting

**Figure 11:**
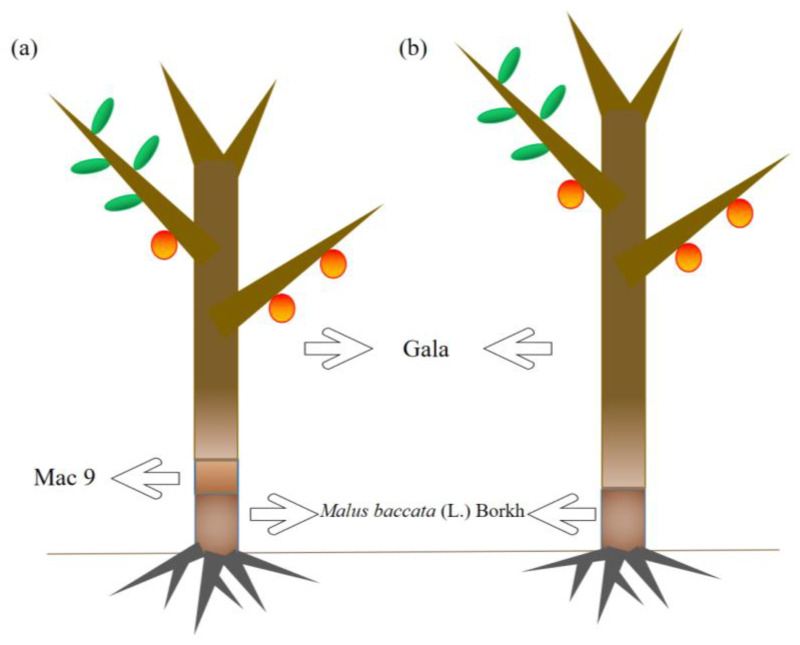
Abridged general view of two grafting combinations. (**a**) ‘Gala’/‘Mac 9’/*Malus baccata* (L.) Borkh and (**b**) ‘Gala’/*Malus baccata* (L.) Borkh.

The experimental samples were collected from the experimental orchard of the Chinese Academy of Agricultural Sciences (CAAS), Xingcheng, Liaoning, China (120°44′ E, 40°37′ N). The rootstock–scion combinations were planted in 2014. The first grafting was performed in August 2012. The second grafting was performed in August 2013. The experimental treatments for grafting were as follows: (1) ‘Gala’/‘Mac 9’/*Malus baccata* (L.) Borkh. (expressed in the form of ‘scion/interstock/rootstock’), abbreviated as M. (2) ‘Gala’/*Malus baccata* (L.) Borkh. (expressed in the form of ‘scion/rootstock’), abbreviated as B. The plant-row spacing was 1 m × 3.5 m. The soil, fertilizer, and water management were as standard; the field management was consistent; and uniformly growing plants were selected for grafting, as shown in Figure 11.

### 4.2. Determination of Tree Growth

To determine the growth of B and M, the interstock diameter, scion diameter, tree height, crown width, and annual branch growth of the B and M combined trees were measured on 21 June 2016. The measurement position of the interstock diameter was 5 cm above the grafting interface, and the scion diameter was measured 5 cm below the grafting interface. We selected three trees with uniform growth for the B and M combination, and repeated the measurement three times for each indication.

### 4.3. Transcriptome Sequencing Analysis

On 21 June 2016, three trees that had developed well and were free of illnesses and insect pests were selected for B and M. A phloem slice of 2 cm in length and breadth was obtained for each sample. Each of the five samples (BS, BR, MS, MI and MR) had three biological replicates. The phloem samples were kept in liquid nitrogen for a short time before being moved to a −80 °C ultra-low-temperature refrigerator. The Trizol technique was used to extract total RNA from the 15 phloem samples. Following purification, the total RNA integrity and concentration were determined. Following the successful completion of the test, a cDNA library was created. The cDNA library was sequenced using the Illumina HiSeq high-throughput sequencing platform (USA), which is based on sequencing-by-synthesis (SBS) technology. The raw data were filtered and quality-regulated, while the Q20, Q30 and GC contents of the clean data were determined. Using TopHat 2 software (v 2.1.1, http://ccb.jhu.edu/software/tophat/index.shtml (accessed on 2 October 2022).), the filtered clean data were compared to the apple reference genome (*Malus* × *domestica* GDDH13 v1.1) (https://www.rosaceae.org/species/malus/malus_×_domestica/genome_GDDH13_v1.1 (accessed on 2 October 2022)) [69]. We obtained the sequence alignment efficiency and sequence location information of the reference genome, in addition to sample-specific sequence feature information. We employed alignment of gene sequences to the transcription factor database to obtain information on transcription factors, and the results were recognized and categorized.

### 4.4. Differentially Expressed Gene (DEG) Analysis

DESeq2 software (v 1.36.0, http://www.bioconductor.org/packages/release/bioc/html/DESeq2.html (accessed on 10 October 2022)) was used to conduct the differential expression analysis. Quantitative analysis was performed using Cuffdiff software (v 1.0, http://cufflinks.cbcb.umd.edu/ (accessed on 10 October 2022)). The expression level of a gene was represented by the expected number of fragments per kilobase of transcript sequence per million base pairs sequenced (FPKM), calculated according to the FPKM values of all the genes in each sample and Pearson’s correlation coefficient (R), and drawn as a heat map. Replicate correlations of samples between or within groups were visually assessed. We screened DEGs with fold change ≥ 2 and FDR < 0.01. The false discovery rate (FDR) was obtained by correcting the *p*-value < 0.05 for the significance of the difference. goCluster software (v 1.0.3, https://bioconductor.org/packages/bioc/1.6/src/contrib/html/goCluster.html (accessed on 12 October 2022)) was used to perform Gene Ontology (GO) (http://geneontology.org/ (accessed on 12 October 2022)) functional enrichment analyses on the differential gene sets, and the KEGG orthology-based annotation system (KOBAS) (v 2.0, http://kobas.cbi.pku.edu.cn/ (accessed on 13 October 2022)) was used to screen the Kyoto Encyclopedia of Genes and Genomes (KEGG) database (http://www.genome.jp/kegg/ (accessed on 13 October 2022)) for enriched signaling pathways, with *p* < 0.05 as the threshold of significant difference. On the basis of the selected reference genome sequence (*Malus* × *domestica* GDDH13 v1.1) [69], the mapped reads were spliced using Cufflinks software (v1.0) and compared with the original genome annotation information to identify the original nonannotated transcription region and investigate the new transcripts and genes of the species, so as to supplement and improve the original genome annotation information. Using a basic local alignment search tool (BLAST) (http://blast.ncbi.nlm.nih.gov/Blast.cgi (accessed on 2 November 2022)), the discovered new genes were compared with the Clusters of Orthologous Groups of proteins (COG) (https://www.ncbi.nlm.nih.gov/COG/ (accessed on 15 October 2022)) databases for sequence alignment; KOBAS software (v3.0) was used to obtain the KEGG orthology results of the new genes, and bio-sequence analyses were performed using profile hidden Markov models (HMMER) (v 3.3.2, http://www.hmmer.org/ (accessed on 16 October 2022)) to obtain the annotation information of the new genes after prediction of their amino acid sequences.

### 4.5. Weighted Gene Co-Expression Network Analysis (WGCNA)

WGCNA was performed using BMKCloud (www.biocloud.net (accessed on 2 November 2022)). All the DEGs (15,555) were used to investigate the genes associated with dwarf traits in the grafted interstocks (Table S11). The module’s minimum number of genes was set at 30, and the similarity threshold value was 0.25. After having filtered the low-expression genes (FPKM < 1), an epigen gene-based connectivity (kME) value was calculated for each gene with every module. The kME was used to assess the value of the effective connectivity between the key genes. We chose genes with kME > 0.7 as module members and used KEGG enrichment analysis.

### 4.6. Real-Time Quantitative PCR Validation

Real-time quantitative PCR (qRT-PCR) was carried out to validate the levels of DEGs from the phloem samples of the B and M combinations [70]. According to the instructions of the Prime ScriptTM RT reagent Kit with gDNA Eraser (TaKaRa, Code No. RR036A), 1000 ng of the total RNA was reverse-transcribed with random primers. The reaction system included 1000 ng of RNA and 2 µL of 5 × PrimeScript RT Master Mix (Perfect Real Time), and RNase-free dH_2_O was added to obtain a 20 µL volume; the reaction conditions were 37 °C for 15 min and 85 °C for 5 s. A total of four random genes were selected from the transcriptome data. Primers were designed with primer premier 5.0 software, and the relevant primers were synthesized by Sangon Biotech (Shanghai, China) (Table S12). The qRT-PCR was performed using TB Green Premix Ex Taq II (TaKaRa, Code No. RR820B). The qRT-PCR aliquot contained 10 µL TB Green Premix Ex Taq II (Tli RNaseH Plus) (2×), 6 µL of ddH_2_O, 2 µL of cDNA, 0.8 µL of each of the forward and reverse primers (10 μM), and 0.4 µL of ROX Reference Day II (50×). The reaction conditions included preincubation at 95 °C for 30 s, followed by 40 cycles at 95 °C for 7 s, 60 °C for 30 s, and 72 °C for 30 s, with melting (65~97 °C, +1 °C/cycle; holding time: 1 s). The levels of the five genes were normalized to qActin. All the PCR assays were performed with three biological replicates. The relative expression levels were calculated using the 2^−∆∆Ct^ method [71].

## 5. Conclusions

The RNA-seq technique was used to evaluate the impact of dwarf interstocks on the scion and rootstock in apple trees. The results showed interstocks caused apple tree dwarfing mainly by affecting the expression of genes related to GA and IAA (*GH3*.9, *PIN1*, *CKI1*, *ARP1*, *GA2ox1* and *GA20ox1*), and they also can regulate tree photosynthesis (*MADH-ME4* and *GAPC* were downregulated) in the scion and promote the expression of C-metabolism-related genes (*AATP1*, *GDH* and *PFK3*), N-metabolism-related genes (*NRT2.7*, *NADH* and *GDH*) in rootstocks. Interstocks can also improve the expression of secondary-metabolism-related genes (*DX5*, *FPS1*, *TPS21* and *SRG1*) related to characteristics such as phenols, aroma, and fruit quality. Additionally, the interstocks acquired early-flowering characteristics by promoting the expression of flowering genes in the scions (*MOF1*, *FTIP7*, *AGL12* and *AGL24*). This study will provide a theoretical foundation for selection and practical application apple dwarfing interstocks.

## Data Availability

The raw sequence data reported in this paper have been deposited in the Genome Sequence Archive (Genomics, Proteomics and Bioinformatics, 2021) [72] in the National Genomics Data Center (Nucleic Acids Res, 2022) [73]., the China National Center for Bioinformation/Beijing Institute of Genomics, Chinese Academy of Sciences (GSA: CRA008505), and are publicly accessible at https://ngdc.cncb.ac.cn/gsa (accessed on 20 October 2022).

## Supplementary Materials

The following supporting information can be downloaded at: https://www.mdpi.com/article/10.3390/ijms24010807/s1.
