# Supplementary material for: Transcriptome Analysis of the Effects of Grafting Interstocks on Apple Rootstocks and Scions
Source: Int J Mol Sci. 2023 Jan 2;24(1):807. doi: 10.3390/ijms24010807 (PMC9821396; doi:10.3390/ijms24010807)
Supplement: Supplementary file 1 [file ijms-24-00807-s001.zip › ijms-2049849-supplementary/ijms-2049849-supplementary tables and figures/Figure S.pdf]

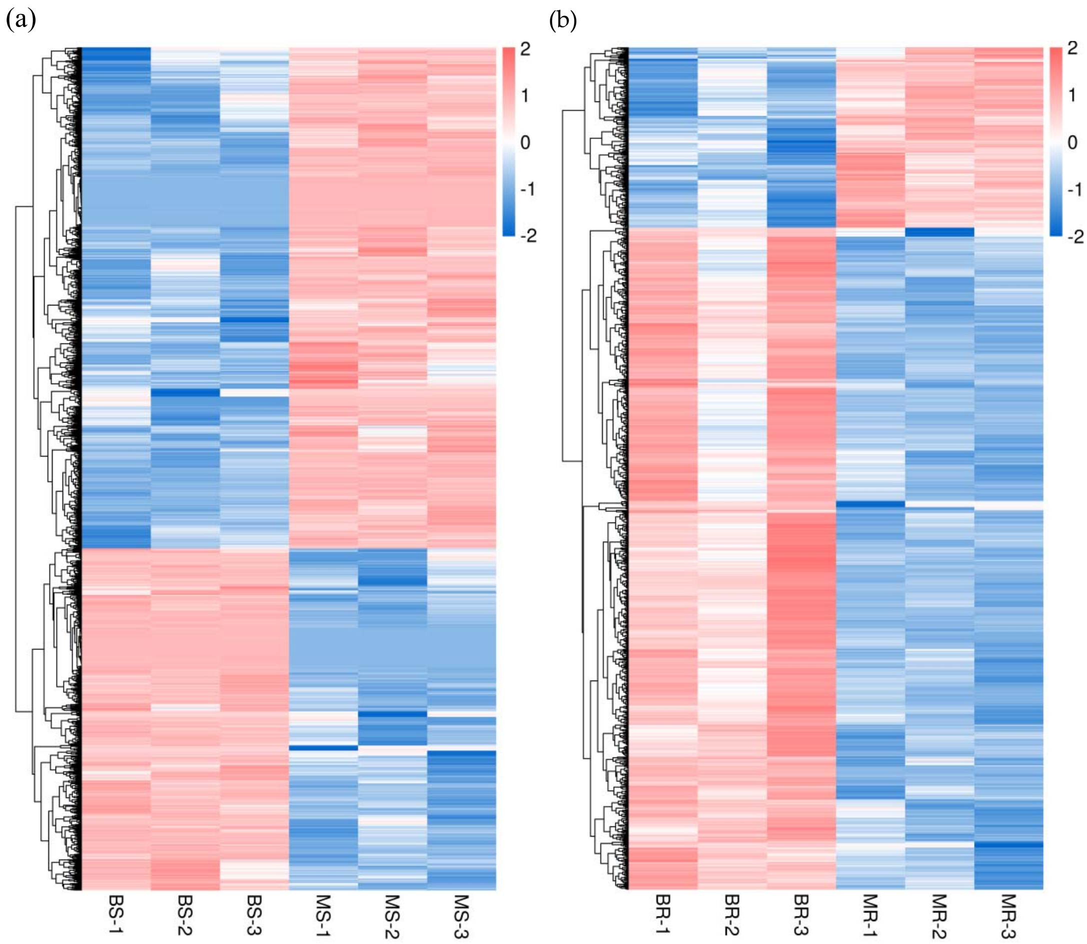

Figure S1 DEGs clustering heat map. (a) BS vs MS DEGs clustering heat map. (b) BR vs MR DEGs clustering heat map. The abscissa represents the sample name and the clustering results of the samples, and the ordinate represents the differential genes and the clustering results of the genes. Different columns in the figure represent different samples, and different rows represent different genes. The color represents the expression level of the gene in the sample (log2(FPKM+1)).

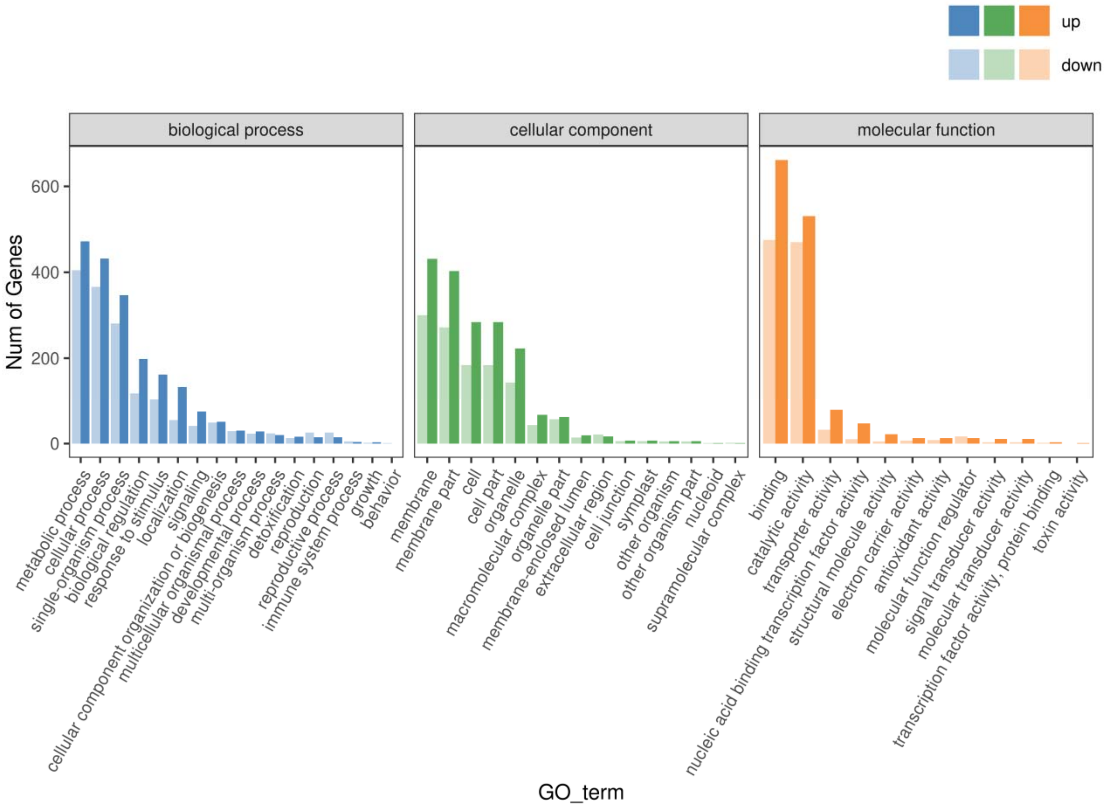
up
down
biological process
cellular component
molecular function
Num of Genes
0
200
400
600
metabolic process
cellular process
single-organism process
biological regulation
response to stimulus
localization
signaling
cellular component organization or biogenesis
multicellular organismal process
developmental process
multi-organism process
detoxification
reproduction
reproductive process
immune system process
growth
behavior
membrane
membrane part
cell
cell part
organelle
macromolecular complex
organelle part
membrane-enclosed lumen
extracellular region
cell junction
symplast
other organism
other organism part
nucleoid
supramolecular complex
binding
catalytic activity
transporter activity
nucleic acid binding transcription factor activity
structural molecule activity
electron carrier activity
antioxidant activity
molecular function regulator
signal transducer activity
molecular transducer activity
transcription factor activity, protein binding
toxin activity
GO_term

(a)

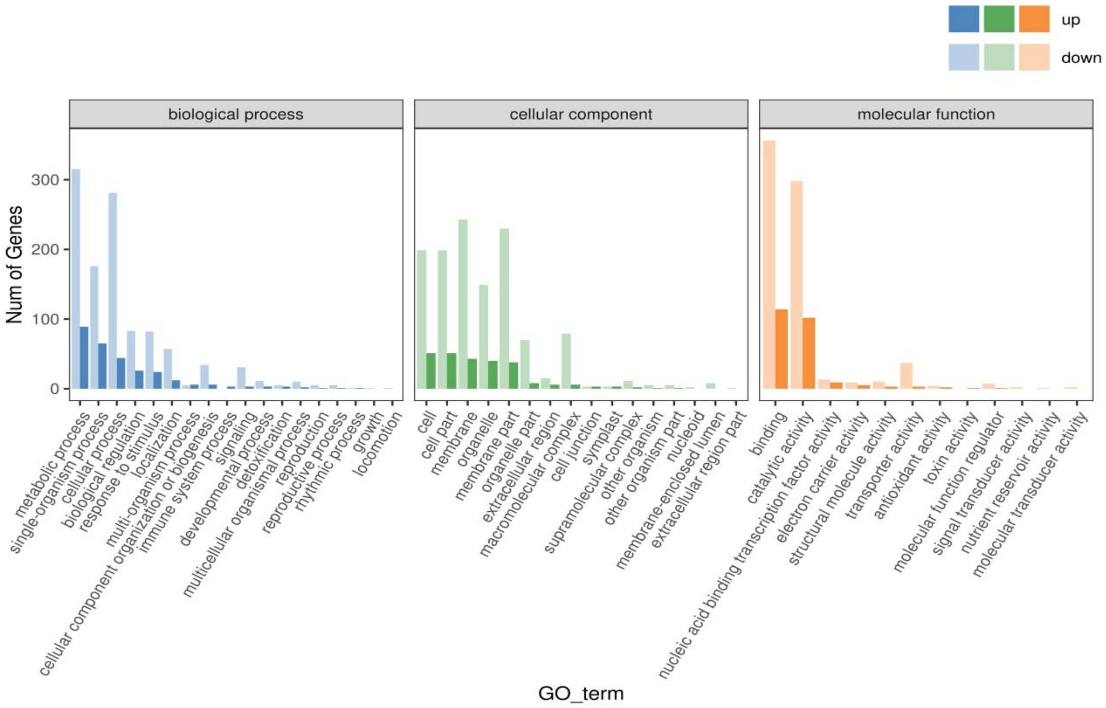
up
down
biological process
cellular component
molecular function
Num of Genes
0
100
200
300
metabolic process
single-organism process
cellular process
biological regulation
response to stimulus
localization
multi-organism process
cellular component organization or biogenesis
immune system process
signaling
developmental process
detoxification
multicellular organismal process
reproduction
reproductive process
rhythmic process
growth
locomotion
cell
cell part
membrane
organelle
membrane part
organelle part
extracellular region
macromolecular complex
cell junction
symplast
supramolecular complex
other organism
other organism part
nucleoid
membrane-enclosed lumen
extracellular region part
binding
catalytic activity
nucleic acid binding transcription factor activity
electron carrier activity
structural molecule activity
transporter activity
antioxidant activity
toxin activity
molecular function regulator
signal transducer activity
nutrient reservoir activity
molecular transducer activity
GO_term

(b)

Figure S2. GO function analysis of DEGs. (a) BS vs MS GO enrichment analysis. (b) BR vs MR GO enrichment analysis. (Y-axis: name of GO terms; X-axis: number of DEGs involved in this term).

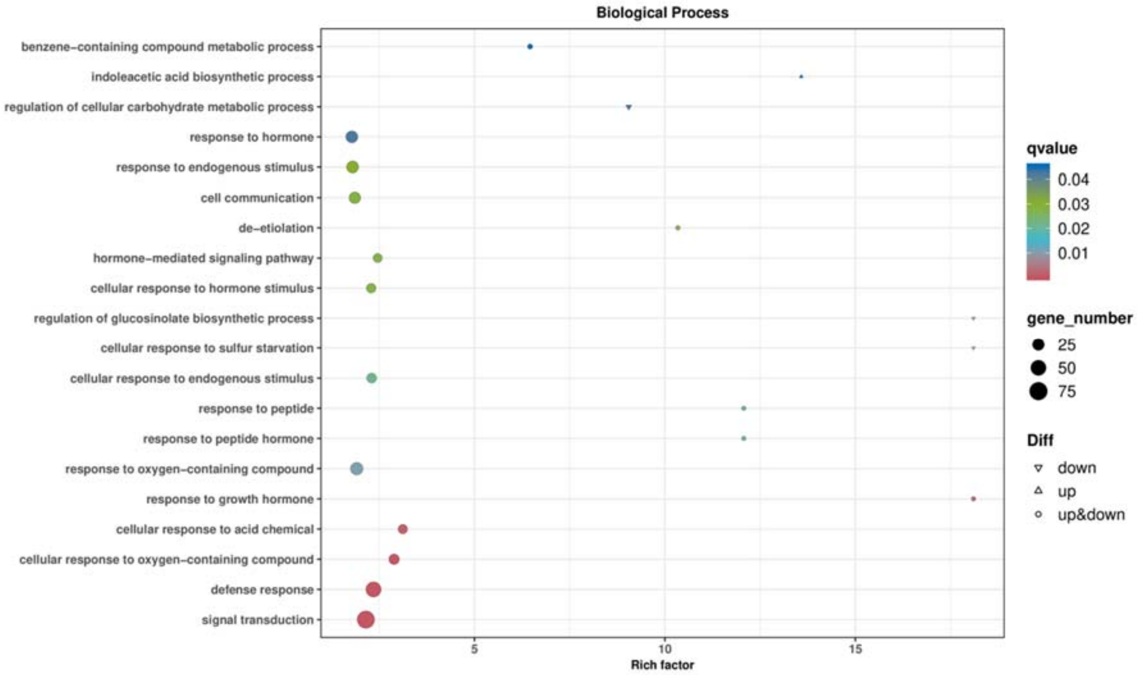

(**a**)

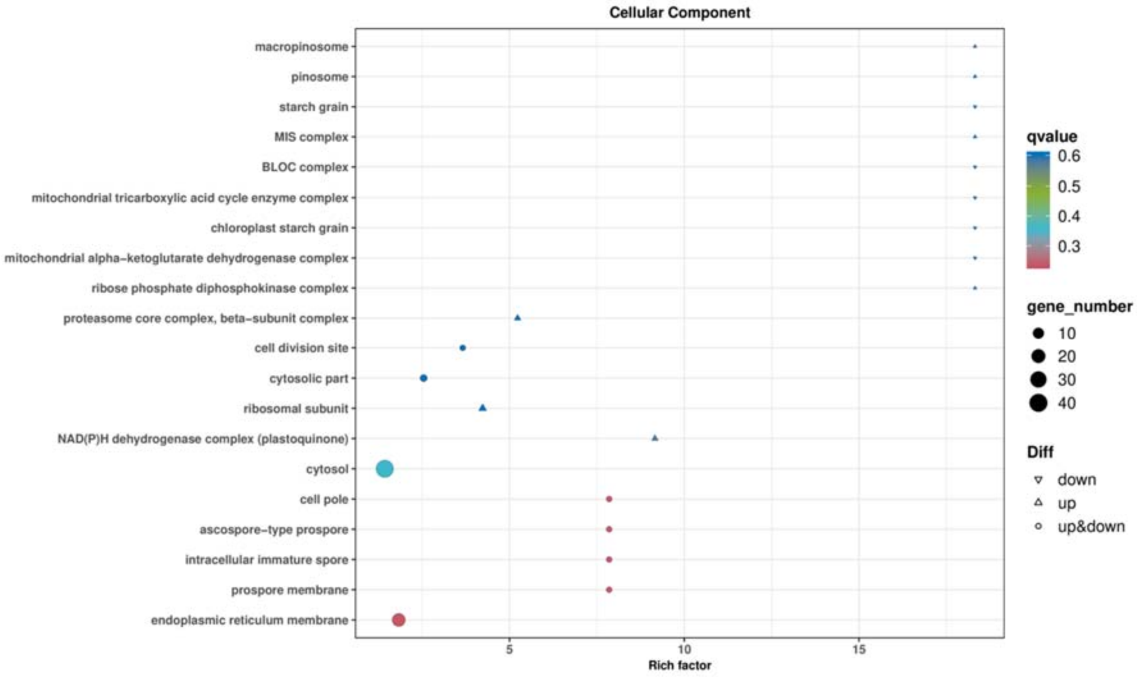

(**b**)

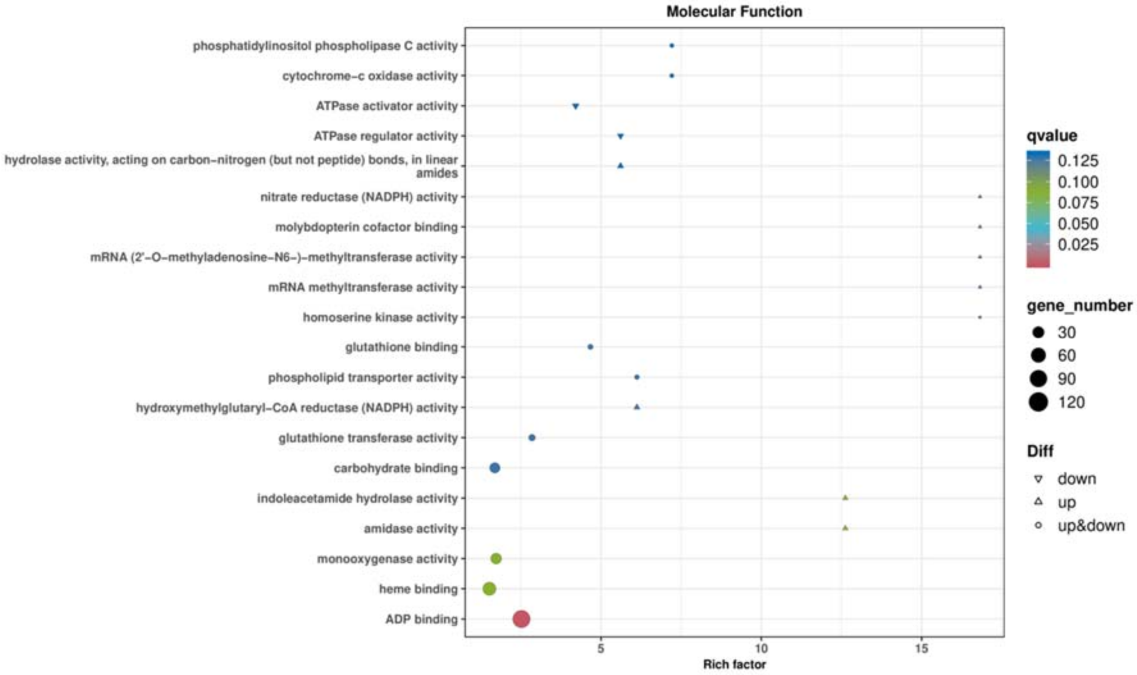
Molecular Function
phosphatidylinositol phospholipase C activity
cytochrome−c oxidase activity
ATPase activator activity
ATPase regulator activity
hydrolase activity, acting on carbon−nitrogen (but not peptide) bonds, in linear amides
nitrate reductase (NADPH) activity
molybdopterin cofactor binding
mRNA (2'−O−methyladenosine−N6−)−methyltransferase activity
mRNA methyltransferase activity
homoserine kinase activity
glutathione binding
phospholipid transporter activity
hydroxymethylglutaryl−CoA reductase (NADPH) activity
glutathione transferase activity
carbohydrate binding
indoleacetamide hydrolase activity
amidase activity
monooxygenase activity
heme binding
ADP binding
5
10
15
Rich factor
qvalue
0.125
0.100
0.075
0.050
0.025
gene_number
30
60
90
120
Diff
down
up
up&down

(c)

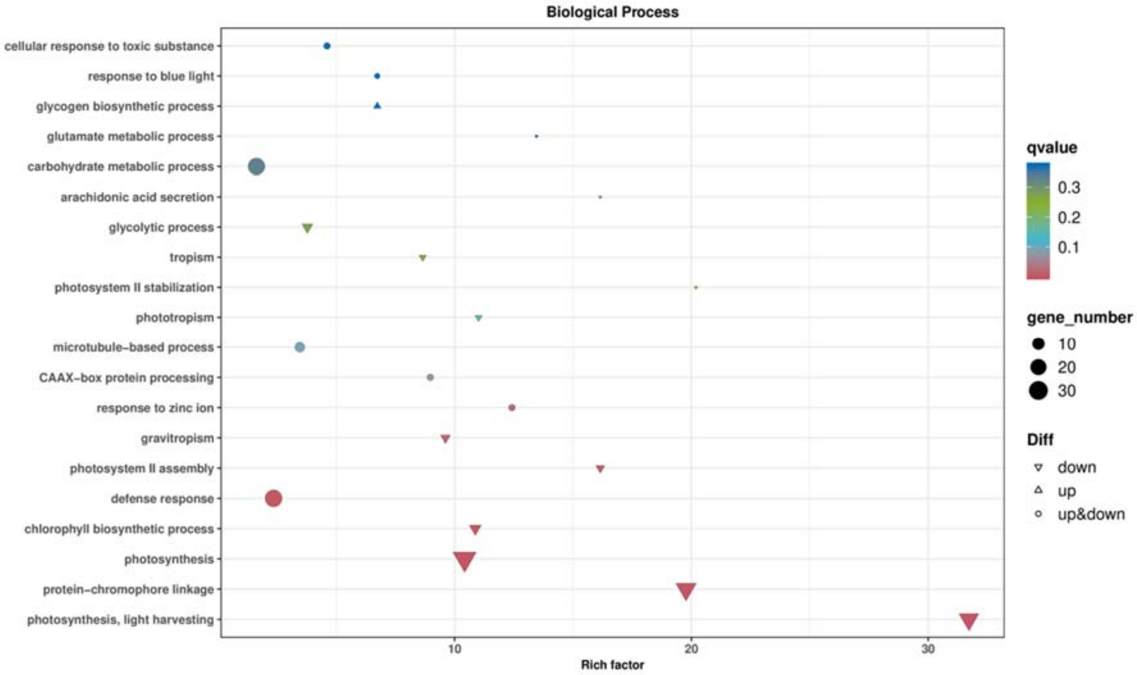
Biological Process
cellular response to toxic substance
response to blue light
glycogen biosynthetic process
glutamate metabolic process
carbohydrate metabolic process
arachidonic acid secretion
glycolytic process
tropism
photosystem II stabilization
phototropism
microtubule−based process
CAAX−box protein processing
response to zinc ion
gravitropism
photosystem II assembly
defense response
chlorophyll biosynthetic process
photosynthesis
protein−chromophore linkage
photosynthesis, light harvesting
10
20
30
Rich factor
qvalue
0.3
0.2
0.1
gene_number
10
20
30
Diff
down
up
up&down

(d)

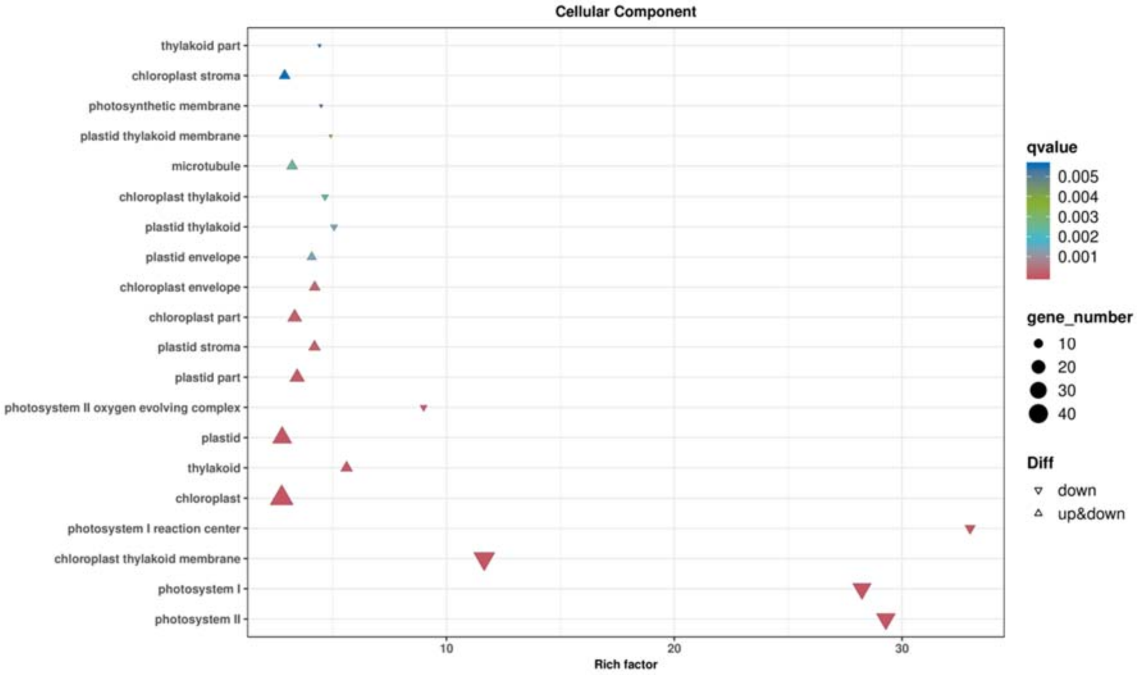

(**e**)

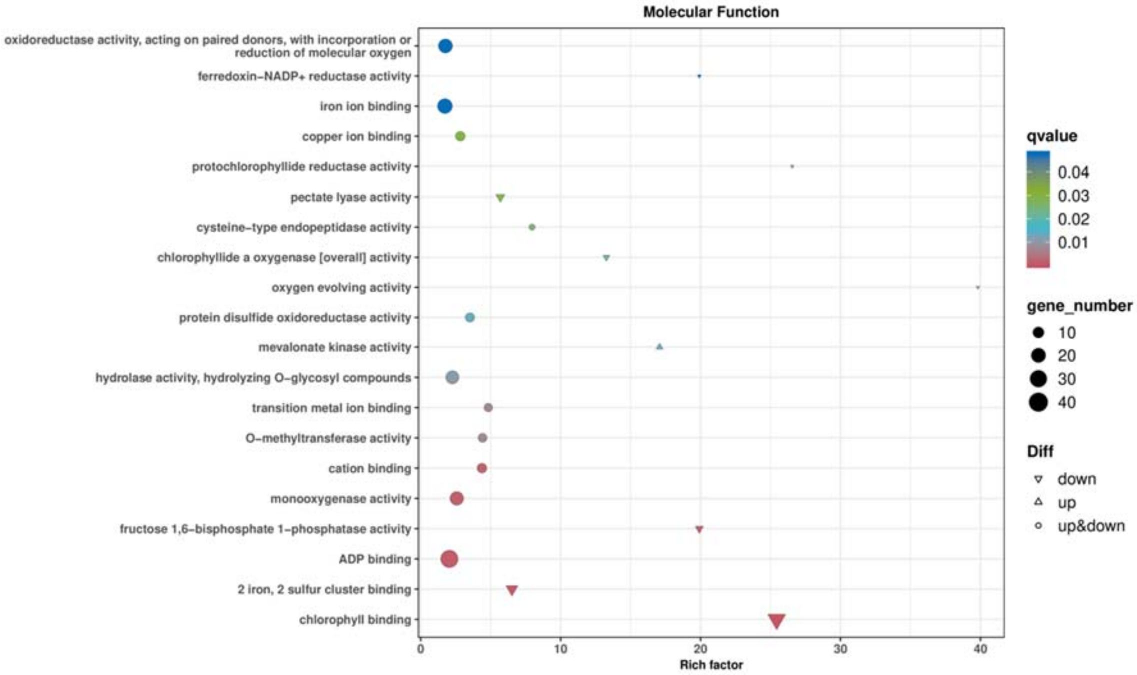

(f)

Figure S3. GO enrichment on DEGs in bubble chart. (a-c) BS vs MS biological process, cellular component, and molecular function in bubble chart. (d-f) BR vs MR biological process, cellular component, and molecular function in bubble chart. (Abscissa was number of DEGs enriched in the GO term, ordinate for each GO team annotation entry. The size of the dots represents the number of DEGs annotated in the pathway, and the color of the dots represents the q-values of the hypergeometric test).

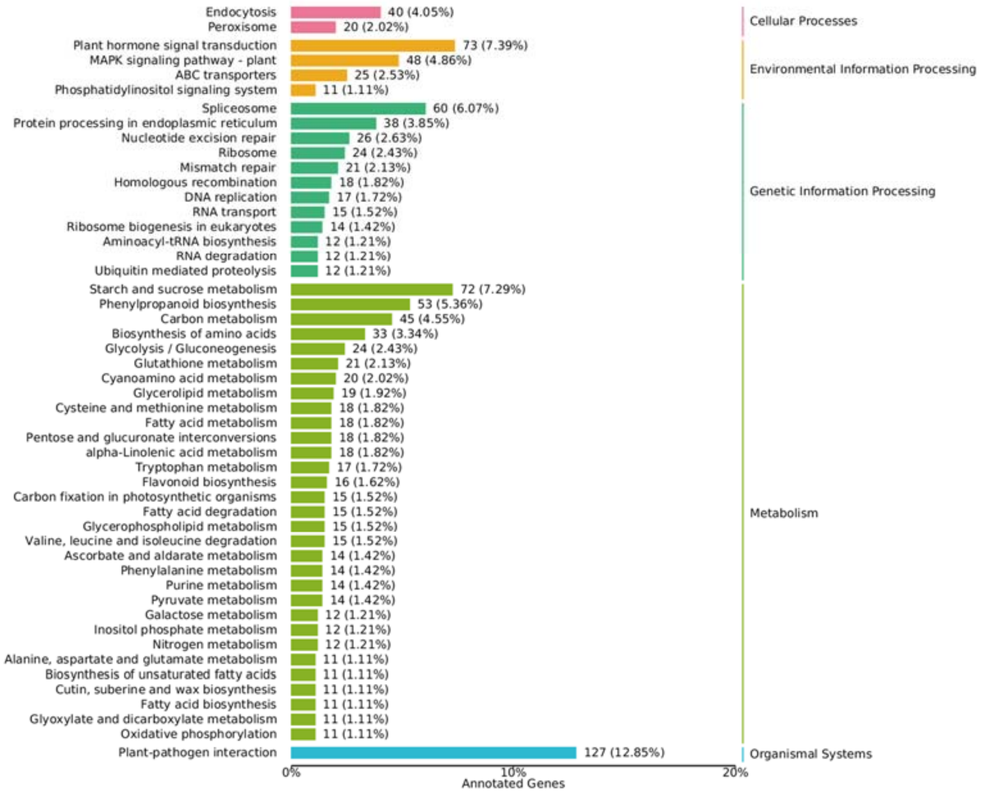

(**a**)

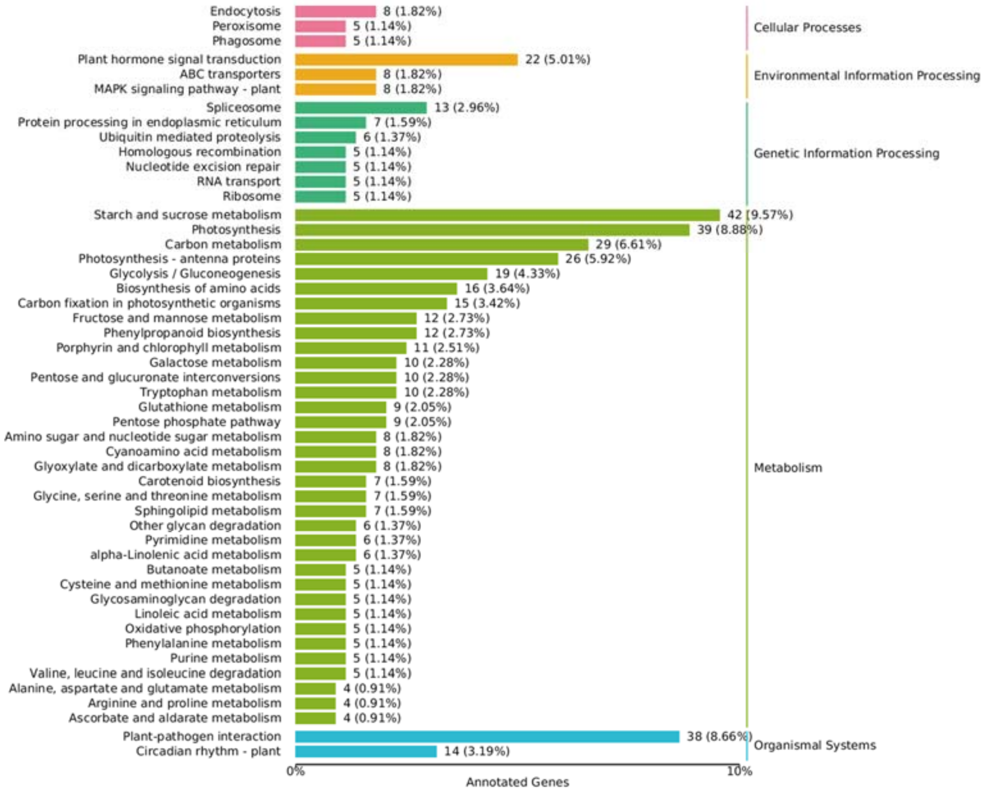

(**b**)

Figure S4. Enrichment analysis of KEGG. (a) BS vs MS KEGG enrichment histogram. (b) BR vs MR

KEGG enrichment histogram. (Abscissa was number of DEGs enriched in the KEGG pathway, ordinate for each KEGG pathway annotation entry).

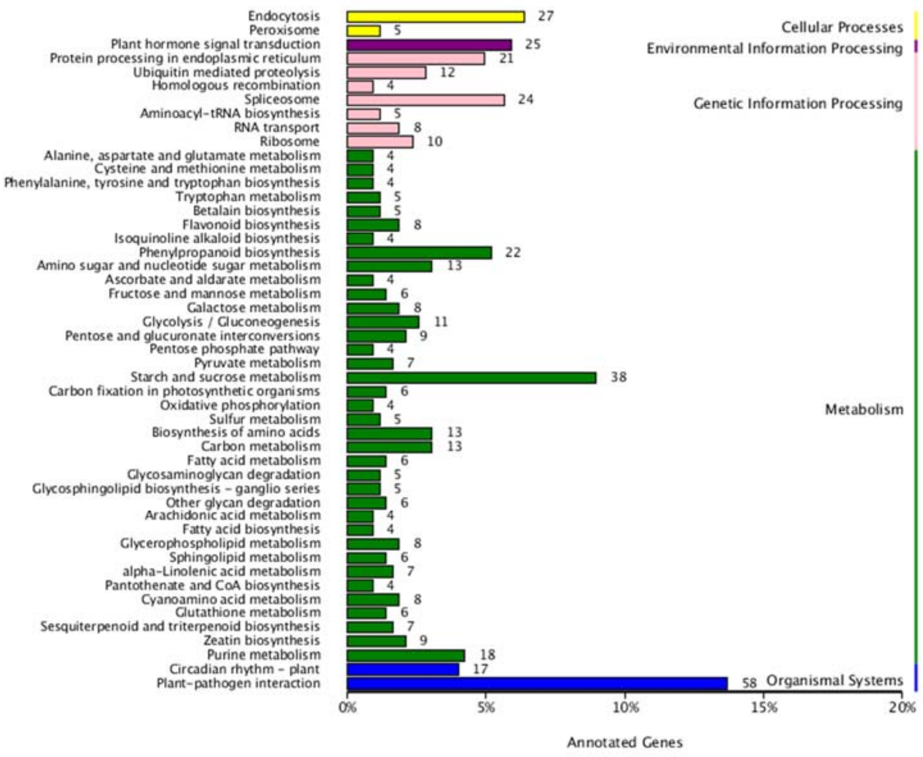

(**a**)

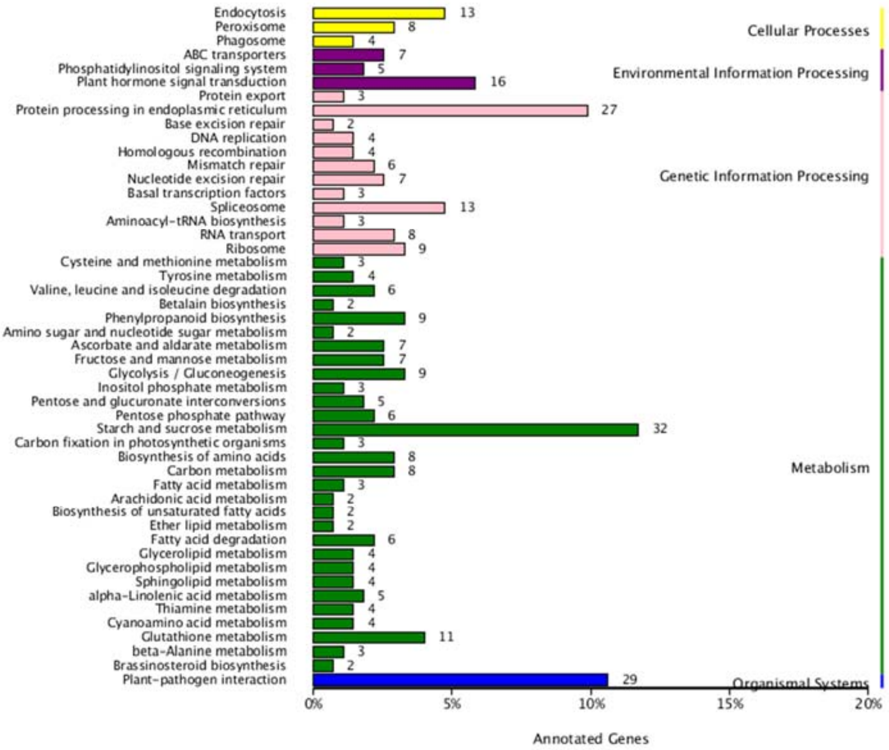

(**b**)

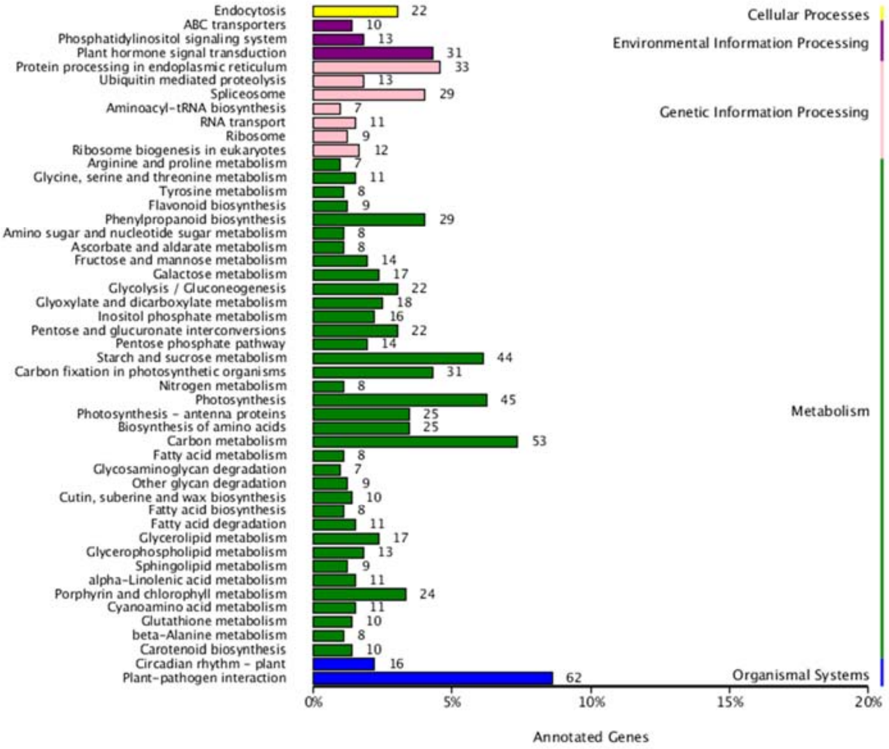

(**c**)

Figure S5. KEGG pathway analysis of the DEGs in each modules. (a) MEblack KEGG enrichment histogram. (b)MEtan KEGG enrichment histogram. (c)MEgreen KEGG enrichment histogram. (Y-axis: name of KEGG pathway; X-axis: number and ratio of genes involved in this pathway over counts of all

annotated genes).

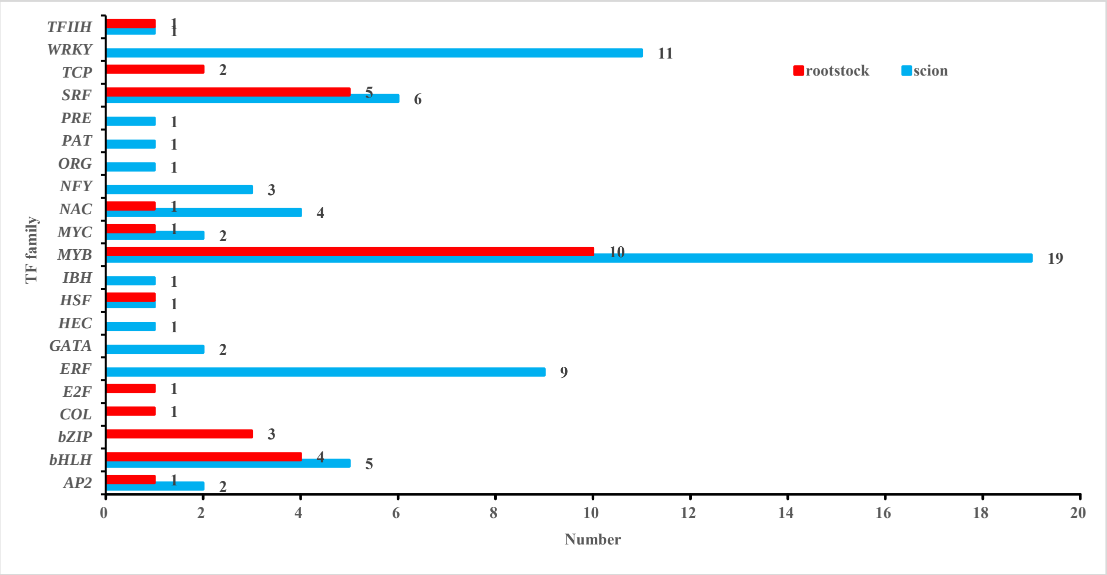

Figure S6. Family analysis of differential transcription factors (DTFs). (Y-axis: name of transcription factors family; X-axis: number of the DTFs involved in this family)
